# [Retracted] MicroRNA-4500 suppresses tumor progression in non-small cell lung cancer by regulating STAT3

**DOI:** 10.3892/mmr.2026.13814

**Published:** 2026-02-02

**Authors:** Zhi-Ying Li, Zi-Zhou Zhang, Hui Bi, Qiu-Di Zhang, Su-Juan Zhang, Lin Zhou, Xiao-Qin Zhu, Jun Zhou

Mol Med Rep 20: 4973–4983, 2019; DOI: 10.3892/mmr.2019.10737

Following the publication of the above paper, it was drawn to the Editor's attention by a concerned reader that certain of the flow cytometric data shown in Fig. 5A on p. 4978 were strikingly similar to data that had either appeared previously in other papers written by different authors at different research institutes, or which had already been submitted for publication.

In view of the fact that the abovementioned data had already apparently been published prior to its submission to *Molecular Medicine Reports*, the Editor has decided that this paper should be retracted from the Journal. The authors were asked for an explanation to account for these concerns, but the Editorial Office did not receive a reply. The Editor apologizes to the readership for any inconvenience caused.

